# The help for people with money, employment or housing problems (HOPE) intervention: pilot randomised trial with mixed methods feasibility research

**DOI:** 10.1186/s40814-018-0365-6

**Published:** 2018-11-13

**Authors:** M. C. Barnes, A. M. Haase, L. J. Scott, M-J Linton, A. M. Bard, J. L. Donovan, R. Davies, S. Dursley, S. Williams, D. Elliott, J. Potokar, N. Kapur, K. Hawton, R. C. O’Connor, W. Hollingworth, C. Metcalfe, D. Gunnell

**Affiliations:** 1 Population Health Sciences, University of Bristol, Canynge Hall, Bristol, BS8 2PS UK; 2 National Institute for Health Research Collaboration for Leadership in Applied Health Research and Care West, UH Bristol NHS Trust, UK/Population Health Sciences,, University of Bristol, Bristol, UK; 3 School of Policy Studies, University of Bristol, Bristol, UK; 4 School of Veterinary Sciences, University of Bristol, Bristol, UK; 5 Public Patient Involvement, Faculty of Health and Applied Sciences, University of the West of England, Bristol, UK; 6 Psychiatric Liaison Team, UHBristol NHS Trust, Bristol, UK; 7 Centre for Suicide Prevention, University of Manchester, Manchester, UK; 8 Centre for Suicide Research, University of Oxford, Oxford, UK; 9 Suicidal Behaviour Research Laboratory, University of Glasgow, Glasgow, UK; 10 NIHR Biomedical Research Centre at the University Hospitals Bristol NHS Foundation Trust and the University of Bristol, Bristol, UK

**Keywords:** Self-harm, Financial hardship, Support intervention, Motivational interviewing, Feasibility

## Abstract

**Background:**

Job loss, austerity measures, financial difficulties and house repossession contribute to the risk of self-harm and suicide during recessions. Navigating the benefits system and accessing sources of welfare and debt advice is a difficult experience for vulnerable people, further contributing to their distress. Whilst there is some evidence that advice-type interventions can lead to financial gain, there is mixed evidence for their effectiveness in improving mental health in those experiencing financial difficulties. There have been no interventions targeting those who have self-harmed due to economic hardship.

**Methods:**

Our aim was to determine the feasibility and acceptability of a brief psychosocial intervention (the ‘HOPE’ service) for people presenting to hospital emergency departments (ED) following self-harm or in acute distress because of financial, employment or welfare (benefit) difficulties. Nineteen people consented to random allocation to the intervention or control arm on a 2:1 basis. Participants randomised to the intervention arm (*n* = 13) received up to six sessions of 1:1 support provided by community support staff trained in Motivational Interviewing (MI). Control participants (*n* = 6) received a one-off session signposting them to relevant support organisations. Fourteen participants were followed up after 3 months. Participants and mental health workers took part in qualitative interviews. The acceptability of outcome measures including the PHQ-9, GAD-7, repeat self-harm, EQ5D-5 L and questions about debt, employment and welfare benefits were explored.

**Results:**

Interviews indicated the main benefits of the service as the resolution of specific financial problems and receiving support when participants were feeling most vulnerable. Randomisation was acceptable to most participants although not always fully understood and control participants could be disappointed. Recruitment was slow (1–2 per month). The outcome measures were acceptable and appeared sensitive to change.

**Discussion:**

The HOPE intervention is feasible and acceptable. There was evidence of need and it is a relatively inexpensive intervention. Refining aspects of the intervention would be straightforward. A full-scale RCT would be feasible, if broadened eligibility criteria led to increased recruitment and improvements were made to staff training and support.

**Trial registration:**

ISRCTN58531248.

**Electronic supplementary material:**

The online version of this article (10.1186/s40814-018-0365-6) contains supplementary material, which is available to authorized users.

## Background

Job loss, financial difficulties and housing problems are associated with an increased risk of depression, self-harm and suicide [1–3] People with pre-existing mental health problems are particularly vulnerable to the effects of financial difficulties [4, 5] and such individuals are also the most likely to lose their jobs during periods of economic recession [6, 7].

Interventions to mitigate the effects of unemployment, financial difficulties and home loss on mental health are an important part of policy responses to these issues, particularly during periods of recession, but the evidence of what works best is limited. A review of studies investigating advice-type interventions, delivered in a range of settings, reported that advice services can lead to financial gain, but there is limited evidence of mental health improvements [8]. These advice style interventions did not involve psychotherapeutic techniques and were given by a range of people including citizens advice volunteers/workers welfare rights officers and advice workers and included giving advice in people’s homes, primary care, via telephones, offices or job centres about benefits, debt, employment, housing, legal issues, amongst many others.

A systematic review of interventions to reduce the impact of unemployment and economic hardship on mental health in the general population found only 11 eligible randomised trials and concluded that ‘job-club’ interventions may be effective in reducing depressive symptoms in unemployed people, whilst there was mixed evidence for the effectiveness of CBT-style interventions [9].

Innovative approaches to addressing the impact on mental health and suicide risk of economic stressors are urgently needed. Our recent qualitative research highlighted that vulnerable individuals, particularly those with pre-existing mental health problems, commonly experience difficulties navigating the benefits system and in accessing available sources of welfare and debt advice [5, 10]. To the authors’ knowledge, there have been no trials of interventions specifically targeting people presenting to the Emergency Department (ED) following self-harm or in acute distress and where financial, employment, welfare benefit or housing difficulties were a contributory factor.

Informed by the findings from our earlier research [5, 10–14] and previous interventions, we developed an intervention for this group of patients. The intervention—known as the HOPE (Help fOr People with money, Employment, benefit or housing problems) service—was a navigator-style intervention, consisting of up to six sessions of 1:1 support provided by community support staff trained in motivational interviewing (MI) methods [15]. The HOPE team helped vulnerable individuals manage their financial-, employment- and benefit-related difficulties and helped them access the available sources of practical and mental health support available in the community with the additional aim of enabling participants to feel more confident in dealing with similar difficulties in the future.

This paper describes the findings of a pilot RCT to determine:

- The acceptability of randomisation
- The acceptability of the content of the intervention and control arms to participants and staff
- Likely recruitment rates to a full trial and identify opportunities to increase recruitment
- Recruitment pathways and optimise these
- Likely loss to follow-up
- Additional training needs of the service providers
- The acceptability and appropriateness of outcome measures (health and economic) and the sample size for a full trial with PHQ-9 as the primary outcomes

## Methods

### Participant recruitment and eligibility criteria

The trial protocol has been published previously [16]. People presenting to the Emergency Department (ED) of a large inner-city hospital in the South West of England following self-harm or with suicidal thoughts, depression and/or in crisis and where financial, employment, welfare benefit or housing problems were cited as contributory factors were identified by the members of the hospital’s liaison psychiatry (LP) team as part of the usual assessment. Self-harm is non-fatal, intentional self-poisoning or injury, irrespective of type or motive or degree of suicidal intent [17]. LP staff described the trial to potentially eligible patients and asked for their consent to allow the research team and the service providers (Second Step https://www.second-step.co.uk/) to contact them after hospital discharge.

The inclusion criteria for the trial were (a) age 18 years or over, (b) self-harmed and/or in psychological distress but not meeting the criteria for referral for secondary mental health care, and (c) financial, employment, welfare benefit or housing problems contributing to distress.

People were excluded if (a) they were in receipt of help from agencies providing similar support to HOPE, (b) they were experiencing a psychotic episode, had thought-disorder or who were unable to give consent, (c) addiction was their primary problem, (d) they were not fluent in English (due to insufficient funding for translation services) or (e) they lived outside of the catchment area for the HOPE service.

It was proposed that patients who had consented to being contacted about the trial were seen within 2 weeks of hospital discharge. At this appointment, the trial was described again, consent was sought, and baseline measures were recorded. After the baseline procedures were complete, consenting participants were randomised.

Participants were followed up 3 months after randomisation.

### Randomisation, allocation concealment and blinding

For the purposes of the pilot, the first two participants were allocated to the intervention; this was to allow any necessary amendments to the service process from worker feedback and to ensure skills learnt by the providers were used as soon as possible after training. Subsequently, once a patient agreed to participate and baseline measures had been recorded, the researcher telephoned the study office, logged the patient in to the study, and received the allocation to intervention or control over the telephone. Allocation was according to a simple random sequence of 12 to the intervention group and 6 to the control group; thus allowing us to gather more evidence on those receiving the intervention than the control group, as well as give the staff greater experience in service delivery, enabling us to make any necessary adjustments to the intervention and follow-up training for the HOPE workers**.**

Neither the participant nor the researcher carrying out follow-up interviews was blinded to the patient’s treatment group.

### The intervention

The intervention was delivered by a team of 6 individuals with a minimum of 2 years’ experience working with people with mental health needs (‘HOPE workers’). HOPE workers received 2 days training in the use of a range of MI methods by two Health Psychologists (AH, AB) with extensive MI expertise. MI is a client-centred, facilitating approach to exploring and resolving ambivalence in order to move towards change [15]. The underlying principle of MI connects to self-determination theory [18]: working with the individual to increase their independence, decision-making and confidence when approaching and dealing with their problems.

Intervention arm patients received up to six 1-hour one-to-one sessions with the same HOPE worker throughout, who used MI methods in the interaction. Sessions took place over a 3-month period in the service user’s home, the Second Step office or a place of the service user’s choosing. Some sessions included travel to other organisations, e.g. debt advice agencies. In the sessions, the HOPE worker (i) discussed the patient’s need and jointly created a support plan, (ii) helped with correspondence/interpretation of official letters concerning state benefits, (iii) gave welfare benefits advice, (iv) supported the patient in accessing key agencies (such as benefits or free debt advice), (v) supported the patient and connected them with other community resources, including mental health care.

Control participants received one session with a HOPE worker immediately following their agreement to join the study—usually in the Second Step offices or service users home—signposting them to relevant support organisations. MI-specific methods were not applied in this session.

### Sample size

We did not formally calculate a sample size target for this feasibility study; we recruited over a set period (May 2016 to February 2017) at an ED in South West England.

### Questionnaire data collection and analysis

Questionnaires were completed by participants prior to randomisation and at the 3-month follow-up. Questionnaire measures included validated measures of depression (the patient health questionnaire (PHQ-9)), anxiety (the general anxiety disorder questionnaire (GAD-7)), quality of life (the Euroqol EQ5D-5 L), financial self-efficacy (financial self-efficacy scale; FSES) and questions about debt, employment, welfare benefits and self-harm [19–22]. The PHQ-9 is designed to measure depression severity; values range from zero to 27; high scores (20–27) represent severe depression. The GAD-7 is designed to measure anxiety severity; values range from zero to 21, with higher scores representing higher levels of anxiety. The EQ-5D-5 L is a generic instrument designed to measure health-related quality of life (HRQoL) with weighted index scores which range from − 0.42 to 1, where higher scores represent higher levels of HRQoL. The FSES is designed to measure financial self-efficacy with scores ranging from 6 to 24, where higher scores reflect a greater belief in a person’s ability to manage their own financial circumstances.

Questionnaire data were analysed using Stata 14.2 (StataCorp, College Station, Texas, US, 2015). We present descriptive statistics (means; standard deviations) in the sample as a whole. We do not make specific contrasts been intervention and control participants in view of the small sample size.

### Qualitative data collection and analysis

Participants and HOPE workers were invited to take part in audio-recorded interviews to determine their views about the research processes, the intervention and outcome measures. Interviews with participants were carried out 3 months post-randomisation, usually in the Second Step (service provider) offices or the participant’s home, with one interview by telephone. Interviews lasted between 20 min and an hour. All interviews were carried out by MB, the main qualitative researcher.

The interviews were transcribed and coded using NViVo software. As the sample size was small, data were analysed as individual case studies which included the context of the participants’ situation, notes from researcher observation, HOPE worker interviews and specific reference to the HOPE worker notes written after each session with a participant. There was particular emphasis on the similarities and differences within and between participants (see Additional file 1: Appendix 2 for an example case study). A case study comparison exercise was used with members of the research team (MB, DG, JD) and an independent researcher (DE) to discuss emergent themes and reach consensus. The names of participants have been changed to preserve anonymity.

### Economic data collection and analysis

An economic analysis was undertaken from the perspective of the NHS. The total cost of the intervention was based on development and delivery (intervention and control group) costs. Data on the time taken to develop the HOPE manuals and training materials were collected retrospectively. The amount of time that HOPE workers spent attending training and refresher sessions was prospectively recorded on time sheets. Similar timesheets were used to record the time trainers spent delivering the training sessions. HOPE workers kept records of the duration and content of each of their contacts with trial participants and any related expenses on purpose-designed forms.

## Results

### Trial recruitment

Between 17 May 2016 and 28 February 2017, the liaison psychiatry team saw approximately 2000 patients. Of these, 197 patients who had self-harmed or presented to the ED in acute distress were screened for eligibility for the HOPE feasibility study. One hundred sixty-one patients were excluded, mainly because employment, financial or benefit difficulties had not contributed to their distress (*n* = 65) or they were already receiving support (*n* = 40). Fifty-six people were also excluded for other reasons. Thirty-six people agreed to be contacted after hospital discharge.

Nineteen patients consented to participation and took part in the trial. Fourteen participants had presented to hospital following self-harm, the remaining five presented in crisis or with suicidal thoughts.

**Fig. 1:**
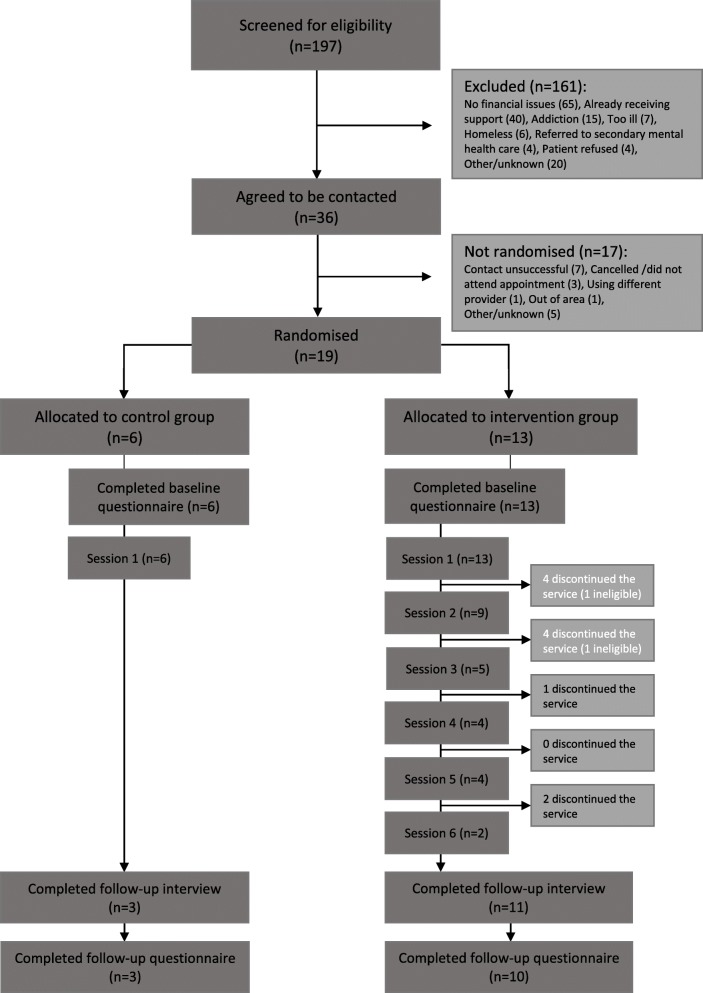
Flow diagram of individuals throughout the pilot study

All 19 randomised patients completed the baseline questionnaire, and thirteen participants were allocated to the intervention group and six to the control group (Fig. 1).

### Participant characteristics

The mean age of participants was 44 years (SD = 9), 11/19 (58%) were male and 18/19 (95%) were white. Most participants were living in rental accommodation (16/19; 84%) and only one participant owned their house outright.

The median time between hospital discharge and the first visit/randomisation was 15 days (range 2–50 days). The mean number of HOPE worker sessions received was 2.8 (range 1–6); only four (31%) of the thirteen intervention arm participants received five or six sessions.

Table 1 shows the state benefits received and financial hardships reported by trial participants. The main welfare benefits received were housing benefit (11/19; 58%), council tax benefit (10/19: 53%), and employment support allowance (5/19; 26%). The main sources of financial hardship and debt were rent arrears (6/19; 32%), utility bills (gas/electricity/water (6/19; 32%), credit card debts (6/19; 32%), loans from family or friends (5/19; 26%) and council tax arrears (5/19; 26%).

**Table 1:** Welfare benefits received and financial hardships reported at baseline and 3-month follow-up

	Baseline		3-month follow-up	
	*n*	%	*N*	%
Benefits				
Number of benefits claimed				
0	4/19	21%	3/13	23%
1–2	5/19	26%	5/13	38%
3–4	10/19	53%	4/13	31%
5+	0/19	0%	1/13	8%
Financial hardships				
Number of payments participant was behind on				
0	2/19	11%	5/13	38%
1–2	10/19	53%	4/13	31%
3–4	6/19	32%	3/13	23%
5+	1/19	5%	1/13	8%

At the 3-month follow-up, interviews were conducted with 3/6 (50%) control arm and 11/13 (85%) intervention arm participants. These three control participants and ten of the intervention participants also completed follow-up questionnaires. Other patients could not be contacted despite several attempts.

### Outcomes

**Fig. 2:**
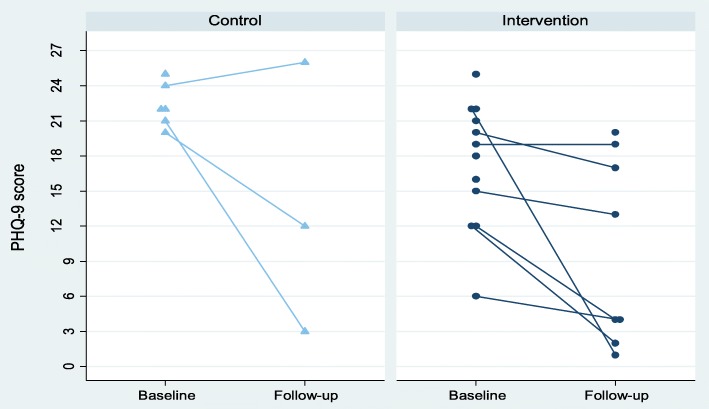
PHQ-9 scores in the intervention and control groups at baseline and 3-month follow-up

The mean PHQ-9 score at baseline (*n* = 19) was 19.0 (SD = 5.1), and nearly two thirds of the participants were categorised as being severely depressed (11/18, 61%). The measures appeared to be sensitive to change, with scores falling by around 40–50% over the follow-up period (see Table 2). Figure 2 illustrates the PHQ-9 scores in the intervention and control groups at baseline and follow-up. The mean PHQ-9 score at the 3-month follow-up (*n* = 13) was 11.0 (SD 8.7); 5/11 participants were without depression and only 2/11 (18%) were categorised as severely depressed. Mean scores for PHQ-9, GAD-7, EQ-5D-5 L and FSES at baseline and follow-up are given in Table 2. Similar scores were seen when only patients who provided data at both baseline and follow-up were included (Additional file 1: Appendix 1).

**Table 2:** Mean (standard deviation (SD)) scores on measures of depression, anxiety, quality of life and financial self-efficacy at baseline and follow-up

	Baseline (*n* = 19)	3-month follow-up (*n* = 13)
PHQ-9*	19.0 (SD 5.1)	11.0 (SD 8.7)
GAD-7	15.0 (SD 4.4)	6.9 (SD 5.9)
EQ-5D^$^	0.76 (SD 0.15)	0.84 (SD 0.19)
FSES^$^	10.1 (SD 3.2)	12.7 (SD 5.1)

Missing data (baseline, follow-up): *(1, 2), ^$^(1, 0)

The main improvements in the FSES occurred in the domains concerning participants’ confidence and perceived the ability to solve financial problems (domains 4 and 5)

Of the 12 participants who answered the self-harm questions at follow up, three had self-harmed in the 3 months following randomisation; all three were in the intervention arm and all stated that they had seriously wanted to kill themselves.

Two intervention arm patients were found to be in receipt of similar services to those offered by HOPE at the first or second intervention session, so they did not receive further input from the team.

#### Qualitative interview findings

##### The acceptability of randomisation

Participants indicated that understanding the recruitment and randomisation process was not a priority amongst their many concerns around the time of their hospitalisation; they viewed HOPE as a service offered to them that could be of some help. Most participants found the post-discharge contact by the HOPE service and randomisation during the first meeting with the HOPE worker and researcher acceptable. However, it was clear during the interviews that the randomisation process and the explanation given to them had not always been fully understood.

How was I told about it? I am not sure actually. I think [HOPE Worker] might have just phoned me up possibly. I cannot really recall. I think some (pause) I do not know. (H09 Control).

That it was going to be about talking (pause) but it was like a trial project but it would be essentially- would be talking to- meeting with [HOPE worker] six times, up to six times, to kind of (pause) discussing and trying to come up with some outcomes and some solutions to what got me in hospital in the first place. (H011 Intervention).

One participant was uncertain about allocation to the full six intervention sessions but was reassured when told they could stop whenever they wanted. Two participants allocated to the control group (a single signposting session) had hoped they would be randomised to the full six intervention sessions; the one participant followed-up had nonetheless appreciated their time with the HOPE worker. There was no evidence of this in the other control case notes. One participant from the control group felt that being randomised to one session meant that his situation was not considered ‘bad’ enough.

##### Acceptability of the content of the intervention and control arm to participants and staff

Most participants in the intervention arm were positive about the HOPE service. They described benefitting from (a) the practical content of the intervention, i.e. help with accessing other support organisations and services, communicating with creditors, and (b) the supportive, enabling and reassuring nature of their relationship with the HOPE worker who had given them extra support at a time when they most needed it and, in some cases, had given them the extra benefit of insight into their coping behaviours.

She’s great, yeah. I have found it quite easy to talk to her, yeah…It wasn’t like sitting down and brainstorming and making mind maps or anything like that (laughs)- it was, yeah, it was just gentle, you know,’ what do you think you should do?’ (H011 Intervention Group).

Basically I buried my head in the sand with it for years and problem’s dealt with now so have not got to worry about it. (H017 Intervention Group).

Feeling that their HOPE worker was on their side, helping them to make their own decisions in a non-judgemental manner could be a powerful experience for some:

What really helped me was the fact that she knew what she was talking about and how people feel in these situations and nudging me… that’s sort of what it’s like, it’s kind of allowing me to sort myself out, almost giving you permission to just- ‘cos she knew that I knew what I needed to do but she didn’t say that I was lazy for not doing it or anything like that… [now] I’m getting up in the morning and thinking ‘ok, what can I do now?’, so I think there’s (pause) there’s ways we can bring in extra money and there’s also ways that we have been spending less, so, that part of it has kind of happened by default, like as though there’s a part of me feeling more like a man in myself, these little things have been taking care of themselves in a way (H015 Intervention Group).

A clear theme from accounts was the value given by service users to the ‘nudge’ from HOPE workers to support them in achieving the small goals they had set together. Often, this was done by texting a reminder to the service user in between face-to-face sessions.

The approach (Motivational Interviewing) used by the service did not suit everyone. Two participants wanted a more directive approach at a time when they were feeling very vulnerable and only had one or two sessions. One of these participants felt the timing of the service would have been better 2 months after her ED attendance, giving her a chance to recover psychologically.

Well I wasn’t mentally strong which I mean if she came round today I’d be like ‘right what I want you to help me with’ (H014 Intervention Group).

Participants reported that they received help from just one or two sessions. Participants could appreciate, for example, being able to talk through a situation such as being on the brink of losing a job or having help to open a bank account.

HOPE workers reported a number of challenges and facilitators in providing the intervention. There were delays in responses from statutory agencies, e.g. from the DWP, once a benefit appeal letter had been sent and the avoidance of sessions on occasions by participants, for example around ending contact with clients:

I think also towards the end of the process, endings are really hard, I think definitely for one of them, one of the women, it was ‘oh do we have to meet’… there’s something like actually ‘I do not want to end this so I am not going to-‘(Hope Worker 2).

Due to slow recruitment, HOPE workers fitted the delivery of the intervention around other aspects of their work for Second Step. All HOPE workers thought that for a full trial, a team of dedicated workers who provided the service more or less full-time would be required to make the intervention work well.

I know that [name] has got to the point where she is tearing her hair out trying to get everybody [HOPE workers] in the same room at the same time, as most are part-time and I do not think we have been able to manage it basically, it just has not happened. So obviously that would be something that we’d have to work a lot harder on, if it went to a bigger trial, to make sure because I think it’s very important that everybody gets a chance to debrief. (HOPE Worker 3).

Factors that helped facilitate the delivery of the service included being flexible in the provision of and spacing of sessions and working with and alongside the service user, giving them autonomy:

HOPE workers liked the structure and rationale of the MI techniques and thought they worked; they were ‘*particularly useful with the service user who was perhaps the most reluctant’*. (HOPE worker 1).

HOPE workers reported a number of benefits for participants; these ranged from debt relief and management plans and contact with Wellbeing Therapies, through to less tangible assistance, such as feeling properly listened to in a respectful manner.

I contacted a bank and the next time we arranged to meet we went along and we opened a bank account – she’s now got a new card and a PIN and access to her own benefits which is brilliant (HOPE Worker 2).

[this service user], yeah I feel like he’s moved forward. In terms of the financial situation because he’s taken ownership and has a plan and he’s working towards that – he just needed time to heal himself and other things before he could get to that (HOPE Worker 1).

The service user feels he’s hit rock bottom …I said ‘you’ve obviously been managing really well and you’re capable, it’s just whether you feel that you’re able and want to [work with me]’, and he did a sort of double take and said ‘I’ve not been called capable for a while’ (HOPE worker 2)

Follow-up interviews were only possible with half (*n* = 3) of the control group. One described a positive experience, indicating that the control intervention and sign-posting had been helpful, one was negative about the content and one remembered little of the session.

I think I was a bit scared and I was a bit scared of opening letters and I thought ‘oh how bad will they be’,, so yeah, she sort of writ it down and said ‘how much do you think you owe on this’ and I’ll say ‘well I haven’t paid that for so many months and this is what it was a month’ so she writ it down and she put it on paper… I thought actually it does not look that bad written down so if I get the courage up to ring them and she said ‘if you do explain I think you’ll find they’re not as bad as you’re imagining’ and I do (H013 Control Group).

She [HOPE Worker] was a decent type. She did give me some letter but I lost it with information on it but I am not very good at picking up on like, you know, organisations and sort of hanging on phone lines and trying to get to the front of the queue, mainly because my survival mechanism is ‘I will stay alive’ and I know there’s lots of people who are in a worse state than me so I am not good at like persistent pursuing, you know, and hanging onto phone lines and dealing with one person after another. I just give up because, you know, kind of (pause) it’s just too much emotional stress. (H09 50 Control Group).

After initial nervousness about how a one-off control session would be achieved, the HOPE workers became comfortable with the control sessions through positive experiences with participants.

I think after the first one or two then I was absolutely fine because I thought ‘OK, this is do-able’ and it can be very contained and it felt really handy to just have either… the sheets, the signposting, we have to physically give them something which felt like a nice thing to do (HOPE Worker 2).

#### Additional training needs of the service providers

Accounts from the HOPE workers highlighted a learning curve in providing the service, with the experiences of recruiting, randomising and providing the intervention for the first few patients feeding into further training and intervention manual development. The majority of the servicer users were seen by two of the HOPE workers as three moved jobs/area early in the study and one had a high workload elsewhere. The training was considered good, but HOPE workers would have valued more practice using MI methods. Three top-up training sessions provided by AH/AB were greatly appreciated.

The main challenges identified by the workers in providing the service were to do with delays in starting to provide the service after training and concern about sessions with clients where they had taken a more interventionist and less self-determination approach due to the service user’s situation, for example:

she [service user] was in a really, really bad way, had no money to buy food, no money to pay bills, and was very much like ‘what are you going to do?’ That was really challenging…this is not what I am meant to be doing…it did feel a little clunky to say ‘have you got any thoughts about what we could do?...so we then and there applied for a crisis loan online (HOPE worker 2).

#### Acceptability of outcome measures

Within the qualitative interviews, the participants reported they did not find completing the questionnaire onerous. Participants with literacy difficulties were talked through the measures (*n* = 3 participants).

#### Health economics

##### Development and delivery costs

Costs of the development and delivery of the intervention to patients in both arms of the trial are summarised in Table 3. The largest cost associated with the development of the intervention was for the development of the training materials and the HOPE manuals (£1170.31). The time required for the two trainers to deliver training to the HOPE workers was another key cost in the development stage of the intervention (£814.66).

**Table 3:** HOPE development and delivery costs

Development costs	Delivery costs	
	Intervention group *n* = 13	Control group *n* = 6
Developing training content and manuals Hours, 30^a^ Cost per hour, £39^a^ Subtotal cost = £1170.31	Contact sessions Hours in total, 58 Cost per hour, £49.52^c^ Subtotal cost = £2872.16	Contact sessions Hours in total, 6 Cost per hour, £49.52 Subtotal cost = £297.12
Delivering training and refresher sessions Hours, 15.5^a^ and 10.5^b^ Cost per hour, £39^a^ and £20^b^ Subtotal cost = £604.66^a^ and £210.00^b^	Travel to contact sessions Hours, 5 Cost per hour, £49.52 Subtotal cost = £247.60	Travel to contact sessions Hours, 1 Cost per hour, £49.52 Subtotal cost = £49.52
HOPE workers attending training^c^ sessions Hours: 33.25 Cost per hour, £12.65 Subtotal cost = £420.61	Administrative work Hours: 21.25 Cost per hour, £12.65 Subtotal cost = £268.81	Administrative work Hours: 3 Cost per hour, £12.65 Subtotal cost = £37.95
HOPE workers attending refresher sessions Hours, 9 Cost per hour, £12.65 Subtotal cost = £113.85	Travel expenses Frequency of journeys, 4^d^ Subtotal cost = £16.00	Travel expenses Frequency of journeys, 1 Subtotal cost = £4.00
Total development cost = £2519.43	Total intervention group cost = £3404.57	Total control group cost = £388.59

^a^The course materials were primarily developed and delivered by a health psychologist. ^b^A research associate provided additional support in the delivery of the course. ^c^Differences in staff costs for training vs. contact sessions (£12.65 vs. £49.52) reflect differences in the Second Step payment schedules. ^d^Most of the contact sessions took place at the Second Step office; therefore, there were very few instances where travel expenses were required, but likely to be some missing data too

The average total delivery cost per person in the intervention group was £262 (£3404.57/13 intervention participants), and in the control group, it was £65 (£388.59/6 control participants). The delivery costs were largely driven by the face-to-face contact sessions between HOPE workers and the study participants. Altogether, HOPE workers spent 64 h in face-to-face contact with clients (£2872.16 + £297.12 = £3169.28). Additional travel to contact sessions, administrative work and travel to work expenses accounted for comparatively little of the delivery cost (£623.88). The majority of the contact sessions were held at the Second Step office; therefore, HOPE workers incurred little or no additional travel costs.

##### Use of NHS services

Self-reported resource use data were available for ten intervention and three control group participants for the 3-month follow-up period. Primary care services were reportedly utilised by 13 (77%) of the participants, A&E was visited by 5 (38%) of participants, three (23%) of participants had outpatient hospital appointments and one (8%) participant was admitted to the hospital. For all these services, the number of times the participants used them was poorly recorded.

##### Proposed sample size for a full trial

The sample size was based on a 5% significance level and 90% power. Using PHQ-9 as the primary outcome measure, the sample size for a full trial to detect a reduction in PHQ-9 score of 0.4 SDs would be approximately 266 (133 in each group). To detect a 0.6 SD difference, a sample size of 120 patients (60 in each group) would be needed. Taking account of the 32% loss to follow-up in the study, these numbers would need to be inflated to 391 and 176 respectively.

## Discussion

### Main findings

Interviews with participants and HOPE workers indicated that there were a number of perceived benefits of the service including resolution of specific financial problems and the provision of support at a time when it was most needed. In some cases, interactions with HOPE workers had given participants insight into their coping behaviours.

Randomisation processes were acceptable to the majority of participants, but not always fully understood or remembered; patients’ vulnerability and potential confusion should be taken into account in a full trial, with the researcher being prepared and sensitive to clarify, explain and reassure about the process. Control participants could be disappointed despite explanations, but the disappointment was managed by the HOPE worker in a contained one-off session. However, qualitative interviews revealed that at least one participant derived benefit from the one-off ‘control’ intervention session. This may dilute the assessment of intervention benefits in a full trial. To overcome this, the control arm intervention might simply consist of the provision of information/signposting sheets.

Most patients randomised to the intervention only received one to two sessions with the HOPE worker, some because this was sufficient, others because they did not find the approach helpful. In a full trial, consideration should be given to delivering the intervention in a stepped approach, with a review after one to two sessions and possibly postponing sessions until the participants felt mentally well enough to benefit from them (this could be discussed at the first session). Furthermore, it was clear that some participants found the control intervention useful, and a number of intervention arm participants only attended one to two sessions, highlighting the need to reconsider the nature of the control intervention. Alongside this, the self-determination aspects of the MI methods were not embraced by all participants. Some service users wanted a more directive approach to their problems when they were at their most vulnerable. It may well be that a flexibility in approach that considers these needs could be written into the manual and training. Some participants felt that the service was provided too soon after their episode of acute distress and may have engaged more if it had been provided after they had time to receive treatment and recover more from their acute distress. However, it is recognised that the time of greatest risk of repeat self-harm is in the days and weeks after an index episode, highlighting the potential benefit of early intervention.

### Study strengths and limitations

Using a combination of qualitative and quantitative approaches, we were able to evaluate the potential value of the intervention and the feasibility of implementing a full trial to assess its acceptability, effectiveness and cost-effectiveness.

Only 13 participants (68%) completed follow-up questionnaires, although it would be possible to use routinely available data, e.g. hospital attendance following self-harm or in distress for some outcomes. It was a limitation that half of those randomised to the control arm of the trial were lost to follow-up; thus, we only had interview and outcome data for three control participants, limiting the comparisons that could be made. While it was possible to interview the majority (11/13) of participants, the diversity and particularity of experiences meant that the narrative case study approach was able to elucidate some important findings in relation to the experience of the intervention but could not reach data saturation. It was also the case that participants in the trial were a small sub-group of all people in the wider population affected by economic/employment or benefit difficulties and there may be greater benefits from intervening prior to the acute distress signalled by hospital attendance and amongst a wider group of potential participants. The study excluded people who were not fluent in English; as such individuals may experience particular difficulties navigating available support systems, intervention funding for a full trial should consider include costs for workers with relevant language skills.

#### Relevance to wider literature and implications

There have been a number of studies of public health and health service interventions designed to mitigate the effects of unemployment, debt or austerity measures in the general population [8, 9, 23, 24]. Most of these have focused on support for people following job loss [24]. Few interventions have investigated the effectiveness of welfare and debt advice. A trial offering a free telephone advice intervention to people in debt, recruited from unemployment offices in England and Wales, showed little evidence of a reduction in anxiety or indebtedness, with only 31% of participants in the intervention group taking up the offer of debt advice [25].

More recently, the DeCoDer trial [26] aimed to assess the clinical and cost-effectiveness of the addition of a primary care debt counselling advice service to usual care for patients with depression and debt. The trial experienced recruitment difficulties (only six patients were randomised) and therefore had limited power to investigate the impact of the intervention on the trial’s primary outcome—depression. Qualitative findings from DeCoDer highlight the complex relationship between debt and depression: ‘the impact of each on the other was compounded by other psychological, social and contextual influences’. This statement is relevant to the HOPE pilot and whether the primary outcome of trials aimed at alleviating financial, employment and housing difficulties should be the impact of the difficulties on their mental health or resolution of participants’ difficulties and their ability to tackle future similar difficulties. Both outcomes are clearly important. It can be argued that feeling less depressed may make people better equipped to resolve financial difficulties. It could also be postulated that better management of financial circumstances facilitates improves psychological health. As health research funding agencies generally require studies to use a measure of health as their primary outcome, our power calculation was based on PHQ-9 scores, though resolution of the person’s practical difficulties (e.g. debt) would clearly be important secondary outcomes and analysis models could assess their role as mediators of changes in mood (and vice versa).

## Conclusions

These findings show that the HOPE intervention is feasible and acceptable and that it is possible to enrol and randomise participants, although careful consideration of the process of obtaining consent and explaining procedures is required. There was clear evidence of need, and the economic findings highlighted the relatively inexpensive nature of the intervention. Refinement of aspects of the intervention would be possible.

The slow pace of recruitment highlights the importance of widening study eligibility criteria to different settings beyond the ED and to people with lower levels of distress. Relaxing the eligibility criteria to include people who already have access to help would probably improve recruitment. The service could also be extended to all patients regardless of whether or not they are referred for specialist mental health aftercare.

The results support the development of a full-scale RCT, if steps are taken to widen eligibility and implement the suggested improvements to the staff training, intervention and control arms. Given the extent of the proposed changes, the impact (and feasibility) of the extended recruitment criteria may need to be piloted before the full trial commences or in the early phases of the trial.

### Patient and public involvement (PPI) in developing the research

A group of three service user advisers with lived experience of job loss, unemployment, financial problems, self-harm and mental health problems helped design the study.

## Additional file

Additional file 1: Appendix 1. Table of mean (SD) scores on measures of depression, anxiety, quality of life and financial self-efficacy, only including patients who provided both baseline and 3-month data. Appendix 2**.** An example of case notes. (DOCX 29 kb)

## Abbreviations

ED: Emergency Department of hospital
HOPE: Help for people with money, employment or housing problems
MI: Motivational Interviewing
NHS: National Health Service (UK)
