# Correction to: The help for people with money, employment or housing problems (HOPE) intervention: pilot randomised trial with mixed methods feasibility research

**DOI:** 10.1186/s40814-018-0374-5

**Published:** 2018-11-28

**Authors:** M. C. Barnes, A. M. Haase, L. J. Scott, M-J Linton, A. M. Bard, J. L. Donovan, R. Davies, S. Dursley, S. Williams, D. Elliott, J. Potokar, N. Kapur, K. Hawton, R. C. O’Connor, W. Hollingworth, C. Metcalfe, D. Gunnell

**Affiliations:** 1 Population Health Sciences, University of Bristol, Canynge Hall, Bristol, BS8 PS UK; 2 National Institute for Health Research Collaboration for Leadership in Applied Health Research and Care West, UH Bristol NHS Trust, UK/Population Health Sciences, University of Bristol, Bristol, UK; 3 School of Policy Studies, University of Bristol, Bristol, UK; 4 School of Veterinary Sciences, University of Bristol, Bristol, UK; 5 Public Patient Involvement, Faculty of Health and Applied Sciences, University of the West of England, Bristol, UK; 6 Psychiatric Liaison Team, UH Bristol NHS Trust, Bristol, UK; 7 Centre for Suicide Prevention, University of Manchester, Manchester, UK; 8 Centre for Suicide Research, University of Oxford, Oxford, UK; 9 Suicidal Behaviour Research Laboratory, University of Glasgow, Glasgow, UK; 10 NIHR Biomedical Research Centre at the University Hospitals Bristol NHS Foundation Trust and the University of Bristol, Bristol, UK

## Correction

Following publication of the original article [1], the authors would like to correct the number of participants in the second paragraph under the heading **Relevance to Wider Literature**.

The sentence currently reads:

The trial experienced recruitment difficulties (only six patients were randomised) and therefore had limited power to investigate the impact of the intervention on the trial’s primary outcome—depression.

The sentence should read:

The trial experienced recruitment difficulties (61 patients were randomised, 32 to the intervention) and therefore had limited power to investigate the impact of the intervention on the trial’s primary outcome—depression.
