# Supplementary material for: The help for people with money, employment or housing problems (HOPE) intervention: pilot randomised trial with mixed methods feasibility research
Source: Pilot Feasibility Stud. 2018 Nov 13;4:172. doi: 10.1186/s40814-018-0365-6 (PMC6233378; doi:10.1186/s40814-018-0365-6)
Supplement: Supplementary file 1 — Appendix 1. Table of mean (SD) scores on measures of depression, anxiety, quality of life and financial self-efficacy, only including patients who provided both baseline and 3-month data. Appendix 2. An example of case notes. (DOCX 29 kb) [file 40814_2018_365_MOESM1_ESM.docx]

**Additional file 1**

**1.**

**Appendix 1: Table of Mean (SD) scores on measures of depression, anxiety, quality of life and financial self-efficacy, only including patients who provided both baseline and 3-month data**

**2.**

**Appendix 2: An Example of Case Notes**

**Includes: HOPE Worker notes on sessions, follow-up interview with participant by researcher and reflective notes by researcher**

**Appendix 1 Table: Mean (SD) scores on measures of depression, anxiety, quality of life and financial self-efficacy, only including patients who provided both baseline and 3-month data**

|  | **Baseline** | **3-month follow-up** |
| --- | --- | --- |
| **PHQ-9 (n=10)** | 17.1 (SD 5.6) | 10.1 (SD 8.6) |
| **GAD-7 (n=13)** | 14.5 (SD 5.0) | 6.9 (SD 5.9) |
| **EQ-5D (n=12)** | 0.79 (SD 0.15) | 0.83 (SD 0.20) |
| **FSES (n=12)** | 9.6 (SD 3.1) | 12.6 (SD 5.3) |

**Appendix 2: An Example of Case-Notes**

**Gary/H011 Case Study: Intervention Arm**

A very gentle, softly spoken 37 year old man who had not worked for 6 months (he had been a town planner). He lives alone in a rental flat was on Jobseekers Allowance, council tax and housing benefits. Had split with his girlfriend in the past year, lost his job and been made homeless in a short space of time. He had overdosed and been referred to HOPE.

PHQ9 Baseline: 15 Follow-up: 13

GAD7 Baseline: 8 Follow-up:6

Previous self-harm: Yes

Reasons for crisis/Self-harm:

Anxiety leading up to court case

Fear of losing flat

Money worries

**Case Notes of HOPE Worker EI**

- Number of sessions: 5
- Hours: 4.40 hours (plus travel)
- Location: office with texts alongside.
- Actions: texting Gary to ‘nudge’ him on planned email to housing benefit (between 1 and 2)

Putting safety plan in place with Gary for night before Court Case (2)

Phone call and email to food bank to request a one-off visit by Gary (4)

Letter to Salvation Army (5 – last session)

**Session 1 (09.11.16):** Gary described himself as well-educated having completed a degree in town planning.

In the past year he had lost his job, split with his partner, lost his accommodation. He stole a computer from a workplace and got 100 hrs community service. He has not felt able to complete the CS because of not feeling able to get out bed on the day it’s been due to start. He has found it difficult to work for the same reason. He was homeless sleeping on couches or nightshelter. He wrote a blog about homelessness which people engaged with. He got given accommodation by Solon Housing. He got an allotment (very positive) and was on a TV show about it. He also volunteers with Incredible Edible Bristol (started by another TV show).

Gary has worked on and off, going on and off JSA and housing benefit. He described having always been bad with money – spending as soon as he gets it for instant gratification. Rent arrears have built up to £1,000, he has an eviction notice and doesn’t want to lose him home or be homeless again.

A letter saying he had to attend court for not turning up for CS. He felt unable to cope and took two overdoses in 2 weeks.

EI felt there was lots of change talk both external (I have to do this or will be evicted) and intrinsic (wants to keep his home). They discussed his plan to send an email housing benefit and any barriers there might be. They agreed that EI would send him a text at midday to ask (in Gary’s words) ‘have you done that email yet?’ and if he was having any difficulty EI could ring him and discuss. Gary wanted to be working again by next year and that he had to complete his CS to do this. He wanted the focus at the following week’s session to be around the court case.

EI felt that Gary found it useful to talk through his situation as he hadn’t shared much with his friends or family.

Gary sent a text on the day to say he had sent the email

**Session 2 (15.11.16)**: They reviewed Gary’s goal to email HB and advise them of the days he was unable to work due to sickness (cc to housing association) – he had a reply and they were looking in to it to see how much he would be owed. Gary said he was feeling much better as he had done all he could do. However, he is going to court Friday re missing the last court date and CS. He was concerned as last time he did not go as he had overdosed the night before. They identified triggers from last time and Gary said he would find it useful to create a plan to keep him busy the day before and that evening. EI used lots of reflections, summaries and questions to check it was a plan he could stick to o his own and had numbers to call if necessary. Gary wanted the focus for next week’s session to be around getting to community service. At SecondStep offices.

**Session 3 (28.11.16):** the appointment had to be changed at EI’s behest to the following week. They met and reviewed the plan. Gary was disappointed as had almost got to court and then panicked on the bus. He called his probation worker to let her know he couldn’t. EI reminded that he had stuck to many aspects of his plan and kept himself safe. Gary talked about his new plan which was to go the police station and hand himself in and was prepared to have to stay in cells overnight (although probation worker had said unlikely). He was motivated to do it so he could move on.

Gary and I discussed an unhelpful GP appointment the week before and barriers and solutions to this. EI suggested asking for a GP who specialises in mental health. She also gave number for Wellbeing Therapies, leaflet for Second Step recovery college and emailed him with details for St Mungos recovery college and MIND Sat monthly workshops. At Second Step offices. Session booked for following week.

Texts were exchanged on the allotted day as Gary had begun his CS. Eventually a date was found for the end of December. **It doesn’t look like this happened.**

**Session 4 (19.01.17):** a day prior to session 4 Gary texted EI to ask if she was able to issue a food voucher as his benefit payment was late. SecondStep manager felt it was appropriate as a one-off but EI was not on site and would have to wait until she was back in the office the enxt day. Left a message with the foodbank as no answer asking if she could make a referral for Gary to visit. EI informed Gary when he arrived of her actions and they both wrote an email to the foodbank explaining that she had no more paper vouchers left. EI also advised Gary that only way to be sure was to go to CAB early to request.

EI felt that the session was used by Gary to reflect on what he had achieved in the month since they last saw each other. He has been attending CS and only has 9 days left. He said he realised he worked better with goals so has been thinking some up: filling in application form for MSc, contacting someone who offered him a job last year who is going to get back to him. Gary had not taken his antidepressant meds for 2 weeks and was feeling like he had more energy (although worried may crash when out of his system). He ran out and hasn’t yet gone back to GP. EI thinks this may be because of disappointing consultation last time. Arrangement made for final appointment the following week. At SecondStep offices.

**Session 5 (25.01.17):** they made a plan at the start to make sure he got what he wanted from the final session. He was still waiting for his benefits to start again after doing 2 weeks work.

Letter to Salvation Army – Gary said they were sometimes able to help with peoples’ energy bills. After discussion EI agreed to write the letter in the session advising that the HOPE service was ending and asking them to discuss with Gary other support services if needed in the future. This was shown to Gary.

EI asked if Gary would like to be signposted to other agencies that might provide support. He did and the following was passed on via email:

- Bristol Wellbeing Service
- MIND advocacy
- Rethink
- St Mungos Floating support
- The Sanctuary
- Bristol Mental Health Job Service

Gary said that he was nervous about going forward. EI reminded and gave affirmations about what he had achieved when she hadn’t seen him for 6 weeks or so (begun and nearly completed CS, had Christmas and talking to people about possible work). Gary thanked her and she wished him well for the future.

**Follow-up Interview**

Gary turned up in his wellies on his way to a voluntary gardening project and with a heavy cold. After talking about the recruitment and randomisation process he described the past week as:

*‘a bit rubbishy. Got some problems with rent arrears. Saturday I had a few drinks and had some quite dark thoughts and sort of got a knife out but then I just realised the stupidity of it all and broke down crying ad phoned my mum, which is something I wouldn’t have done before…so then I went and spoke to Talking Money and they’re in the process of trying to sort it out for me this morning [and] I woke up feeling a little better that it’s possibly matters in hand…I knew of them but haven’t used them before but again it’s one of Emma’s recommendations’*

He thought that he probably felt down because he hadn’t spoken to anyone properly for a while compounded by his rent arrears.

He talked about short-term private renting with a social landlord and the fact that he wasn’t 100% sure he wanted to go into town planning again. Maybe something related but although he feels he could get an office job fairly easily, it had made him very miserable before and would do so again.

**HOPE Service**

**Logistics**

He felt the first 3 or 4 sessions were great but that the long break over Christmas were not particularly helpful (they couldn’t get their diaries to match up) and that was partially his fault. He described EI as being pretty flexible on times

**Relationship**

*‘She’s great. I have found it quite easy to talk to her’.* Although that ‘quite’ is interesting – maybe because he doesn’t tend to talk to anyone about his problems or maybe because their personal dynamics.

**Content**

He described the sessions as talking about how he was feeling and what was worrying him and then being encouraged to come up with ideas about how I would deal with the issues.

*Say dealing with money problems…we’d arrange that perhaps I needed a kick up the bum to sort of contact them [housing benefit] the next day so we’d arrange that she would send me a text just to find out if I’d done it or remind me to do it…just having the support really of someone who would listen to what I was saying and then say ‘well what do you think you should do?’ and I’d go well I know what I should be doing ‘cos it’s common sense really but it’s just having that kind of little gentle nudge’*

When asked Gary explained about the housing benefit situation which had led to his rent arrears and OD. He said that it was a 20 minute straightforward job (to re-contact the housing benefit agency) but in his head had built up to a Herculanean task so he needed the ‘prod’.

He also talked briefly talked about the breach of his CS which he described as having ‘*resolved itself as well. That was a case of I eventually just had to go to court and deal with it’*.

He described talking Money as great, a real business like outfit ‘ (laughs) *but I think it’s what I needed’*. The feeling that it was slightly different from his expectations (bleeding heart agency) but the professionalism was (he seemed surprised) just what he needed.

**Mechanism**

He described EI and him talking through the positives of getting the CS done and no longer being in his life and then being able to focus again. ‘*Straightforward stuff – you just need that angel voice speaking out against the devil voice’.*

He also described it as all being gentle, with questions and suggestions about what he could do.

**Evaluation**

He thought it was great and helpful.

*‘What I got most out of the service was having the gentle prods actually happening – just talking about it and then having it in my head – going ‘Oh OK, that actually doesn’t make sense’*.

He described this sort of support more helpful that a list of agencies.

**Help-Seeking**

However, he was struggling with the service ending and having the safe space to talk to someone about his problems:

*I just wish it* [HOPE Service] *was longer to be honest ‘cos I’m not quite sure what to do next really. I just find I’m not very good at talking to friends and family about it (pause) like a deep shame you know, stops me from doing it really which I know is silly but it’s there. Plus with friends and stuff when I do go and see them I don’t want it to be about – I want it to be – I’d like to keep my friends in a positive little circle*

Nevertheless, he had phoned his mother in the previous few days when he was ‘in a tizz’ which had helped. It is not that he is feeling sad about anything in particular, just that he feels sad and needs someone to talk him down.

**Context and Mental Health**

Gary talked about suffering from depression and feeling that he was probably a bit bipolar as he has *control issues (laughs) overspending, over doing, over drinking like when I’m up but then I’ll also have weeks when I can’t get out of bed.*

He talked about having the weirdest year of his life; first losing job, relationship, home and then having the highs of two television shows. Once that had all finished and he got the letter about the breach of CS (he had sent a sick note about his depression making it difficult to get out of bed) he ‘freaked out’ and thought he was going to prison which is when he overdosed.

**Other Support**

Gary talked at some length about the problems he had with his GPs. Essentially he felt unlistened to and unsupported. He was well aware of the benefits of exercise and so on (he had run the London Marathon in 2014) when told to try that, ultimately feeling her advice for him ‘*to get out of the depression cycle is just get out of the depression cycle’*. After the self-harm Gary spoke to a GP on the phone who was very good and offered him a 30 minute consultation. He was very hopeful that this would enable him to talk through his problems properly and maybe get referred on. However the consultation lasted just 7 minutes and once it was established he was taking his medication and was ‘ok today’ that was that.

He would like to be able to ‘escalate’ this situation and talked with EI about techniques about how he could do that – he recognised it as a case of having confidence and sticking to his guns when asking for mental health support at the GP. He had researched the surgery online and there were no GPs who specialised in mental health.

**Future**

He talked in a positive way about his plans for the future once his CS was finished. He was thinking about doing a Masters in applied ecology (being on the ‘good’ side of town planning and saving the butterflies). He is also continuing with his edible gardening volunteering, plus carrying on with his own allotment.

**Reflections**

Gary’s account closely reflected EI case notes, although both were a little hazy about just how much of a gap there was in the service over Christmas. Unlike H05, Gary did not describe the break as helpful – although as pointed out by EI he had managed to start and nearly finish his CS in that time. Gary’s account also reflected anxiety about the service ending which he feels fairly isolated.

He felt it was helpful because: he has self-knowledge and some understanding of how he responds to situations, he therefore appreciated the ‘nudges’ to do things he knows he is perfectly capable of doing; he did not talk to friends and family about his feeling/worries and therefore having EI helped. His ‘Other support’ was less effective – it sounded like he needed some better help from his GP to be able to improve long-term and was, perhaps, on the wrong medication. He did, however, have plans for the future.

Keeping the ‘sad’ side of himself from his family and friends cuts him off from precisely that ‘nudging’.

Whist he very much appreciated EI and the service and felt it very helpful, his PHQ9/GAD on the day did not reflect this. As he said, he had had a bad week. The questionnaire and the interview together can give a fuller picture.

Interesting how he talks about the CS breach as having resolved ‘itself’ rather than the work it took between them. Also as EI being the angel voice speaking out against the devil voice of his inaction/avoidance.
